# Instance-level phenotype-based growth stage classification of basil in multi-plant environments

**DOI:** 10.3389/fpls.2025.1707985

**Published:** 2025-11-18

**Authors:** Jung-Sun Gloria Kim, Soo Hyun Shin, Soo Chung

**Affiliations:** 1 Department of Biosystems Engineering, Seoul National University, Seoul, Republic of Korea; 2 Integrated Major in Global Smart Farm, Seoul National University, Seoul, Republic of Korea; 3 Research Institute of Agriculture and Life Sciences, Seoul National University, Seoul, Republic of Korea

**Keywords:** smart agriculture, controlled environment agriculture (CEA), vision-based phenotyping, automated decision pipeline, BBCH scale

## Abstract

Climate change, shrinking arable land, urbanization, and labor shortages increasingly threaten stable crop production, attracting growing attention toward AI-based indoor farming technologies. Accurate growth stage classification is essential for nutrient management, harvest scheduling, and quality improvement; however, conventional studies rely on time-based criteria, which do not adequately capture physiological changes and lack reproducibility. This study proposes a phenotyping-based and physiologically grounded growth stage classification pipeline for basil. Among various morphological traits, the number of leaf pairs emerging from the shoot apex was identified as a robust indicator, as it can be consistently observed regardless of environmental variations or leaf overlap. This trait enables non-destructive, real-time monitoring using only low-cost fixed cameras. The research employed top-view images captured under various artificial lighting conditions across seven growth chambers. YOLO automatically detected multiple plants, followed by K-means clustering to align positions and generate an individual dataset of crop images–leaf pairs. A regression model was then trained to predict leaf pair counts, which were subsequently converted into growth stages. Experimental results demonstrated that the YOLO model achieved high detection accuracy with mAP@0.5 = 0.995, while the A convolutional neural network regression model reached MAE of 0.13 and R² of 0.96 for leaf pair prediction. Final growth stage classification accuracy exceeded 98%, maintaining consistent performance in cross-validation. In conclusion, the proposed pipeline enables automated and precise growth monitoring in multi-plant environments such as plant factories. By relying on low-cost equipment, the pipeline provides a technological foundation for precision environmental control, labor reduction, and sustainable smart agriculture.

## 1 Introduction

Climate change, shrinking arable land, urbanization, and labor shortages in agriculture have emerged as major threats to the stability of crop production (Lehman et al., 2015; Rathor et al., 2024; Van Delden et al., 2021). In response, indoor farming systems that combine artificial lighting with environmental control technologies are increasingly recognized worldwide as next-generation agricultural solutions (Avgoustaki and Xydis, 2020; Sowmya et al., 2024). The integration of artificial intelligence (AI)-driven control approaches, including machine learning, the Internet of Things (IoT), and computer vision, has substantially enhanced the efficiency of crop monitoring, nutrient management, disease detection, and environmental regulation (Duguma and Bai, 2024; Lovat et al., 2025; Rathor et al., 2024).

Accurate growth stage classification plays a critical role in crop management, including nutrient management, harvest scheduling, and quality improvement (Darlan et al., 2025; Ober et al., 2020). However, most previous studies have relied on time-based criteria such as days after sowing (DAS) or transplanting (DAT), which fail to capture physiological changes and often yield inconsistent results across environments and cultivars (Kim et al., 2025; Loresco et al., 2018; Nadeem et al., 2022; Su et al., 2021). Such arbitrary time divisions reduce reproducibility and may cause discrepancies between actual plant status and assigned growth stages, ultimately leading to suboptimal decisions (Onogi et al., 2021; Rauschkolb et al., 2024).

In contrast, growth stage classification based on phenotypic traits offers a physiologically meaningful and reproducible alternative. However, most vision-based approaches developed to complement time-based staging have relied on predicting biomass or leaf area. In particular, data acquisition has been constrained by approaches that photograph or physically measure individual pots (Elangovan et al., 2023; Singh et al., 2023), which are labor-intensive and potentially damaging to plants (Boros et al., 2023; Golzarian et al., 2011). To address these issues, fixed rail systems have been introduced to capture top-view images automatically (Kim et al., 2024); however, they require substantial installation costs, large physical space, and continuous maintenance under humid conditions, limiting their scalability in compact plant factory environments. Moreover, most prior research has ultimately depended on single-plant images or simplified multi-plant data by reducing them to single-plant representations, such as selecting only the largest contour for analysis (Bashyam et al., 2021; Teimouri et al., 2018; Yang et al., 2025).

Therefore, for practical application in large-scale cultivation, it is essential to develop vision-based predictive models that can automatically determine growth stages without relying on direct human observation (Jin et al., 2020). Such approaches enable real-time acquisition of growth information while remaining non-destructive to the plants (Buxbaum et al., 2022). Indicators such as biomass or leaf area are highly sensitive to lighting conditions, camera installation height, and viewing angle, which can lead to discrepancies between measured values and the actual developmental stage (Adedeji et al., 2024). For instance, under red-light conditions, plants often exhibit excessive elongation, making them appear larger and more developed in images, even though their true growth stage has not advanced. Consequently, vision-based metrics are prone to distortion and have proven unreliable as robust indicators for stage classification. On the other hand, the Biologische Bundesanstalt, Bundessortenamt, and Chemical Industry (BBCH) scale provides a standardized framework for describing crop development, from germination through senescence, using discrete morphological indicators such as leaf number (Bleiholder et al., 2001). Adaptations of the BBCH scale have been successfully applied to Arabidopsis, tomato, and basil (Boyes et al., 2001; Cardoso et al., 2021; Stoian et al., 2022). Yet, these studies primarily focused on manual observation, which is labor-intensive, prone to observer bias, and unsuitable for integration into automated environmental control systems. Leaf-pair number at the shoot apex represents a more stable and discrete trait for growth stage classification. It can be consistently observed under diverse environmental conditions and is robust against overlapping leaves or shading. Moreover, fixed cameras capturing top-view multi-plant images provide a cost-effective and scalable means for continuous monitoring, yet such data have not been effectively exploited for automated instance-level stage classification.

In this study, we propose a novel framework for automated, instance-level growth stage classification in multi-plant environments, thereby enabling physiology-based instance-level monitoring from top-view images in fixed-bed cultivation systems. Specifically, we propose: (i) an automated pipeline for large-scale dataset construction using You Only Look Once version 8 (YOLOv8)-based object detection and coordinate ordering in a consistent sequence, (ii) a phenotype-based classification model aligned with the BBCH scale, and (iii) validation of its accuracy and reproducibility across diverse cultivation environments. By anchoring growth stage determination to a physiologically stable trait and integrating it into a scalable detection–ordering–regression pipeline, this study enables non-destructive, real-time stage classification and establishes a foundation for precision control, labor reduction, and sustainable smart agriculture.

## 2 Materials and methods

### 2.1 Top-view image dataset from growth chambers

**Figure 1:**
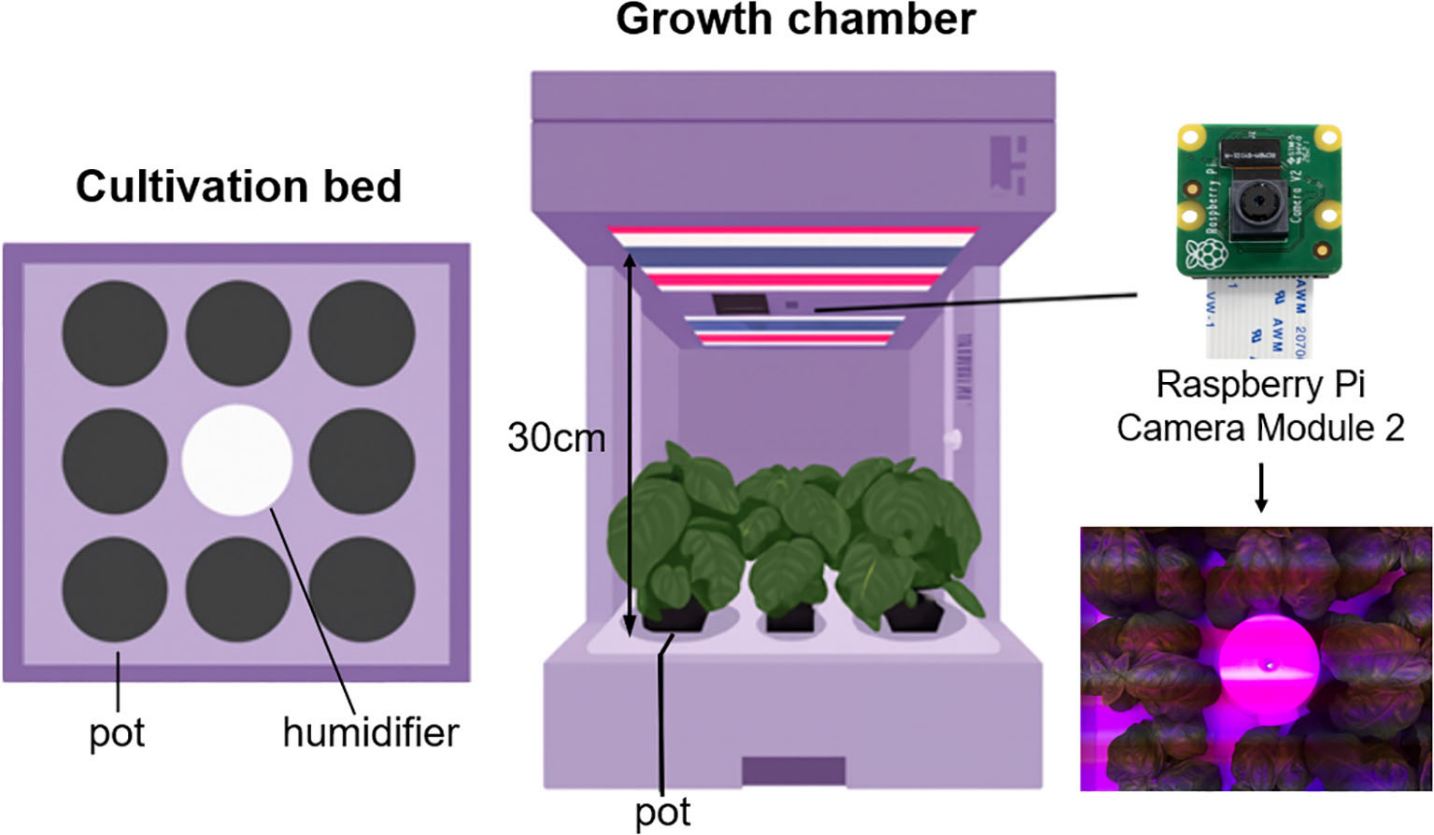
Overview of the top-view image collection system in growth chambers.

As shown in **Figure 1**, a total of nine growth chambers were used in this study, and the distance between the bed and the camera of each cultivator was fixed at 30 cm. A single humidifier was placed at the center of each cultivation bed, and eight pots were arranged around it. Cultivation was conducted under different light conditions depending on the growth stage, and each chamber was set with varying combinations of wavelength. The images captured under these conditions reflect diverse growth environments.

Data was collected from April 20 to May 31, 2024. A total of 8,519 images were collected from nine growth chambers under varying light conditions (**Table 1**). The differences in the number of images among chambers were attributed to variations in the start and end times of operation for each device.

**Table 1:** Number of collected images per growth chamber (April 20–May 31, 2024).

Growth chamber no.	Number of images
1	945
2	947
3	945
4	945
5	943
6	946
7	957
8	954
9	937
Total	8,519

All captured images were stored at a resolution of 3280 × 2464 pixels.

### 2.2 Target crop and growth stage definition

**Figure 2:**
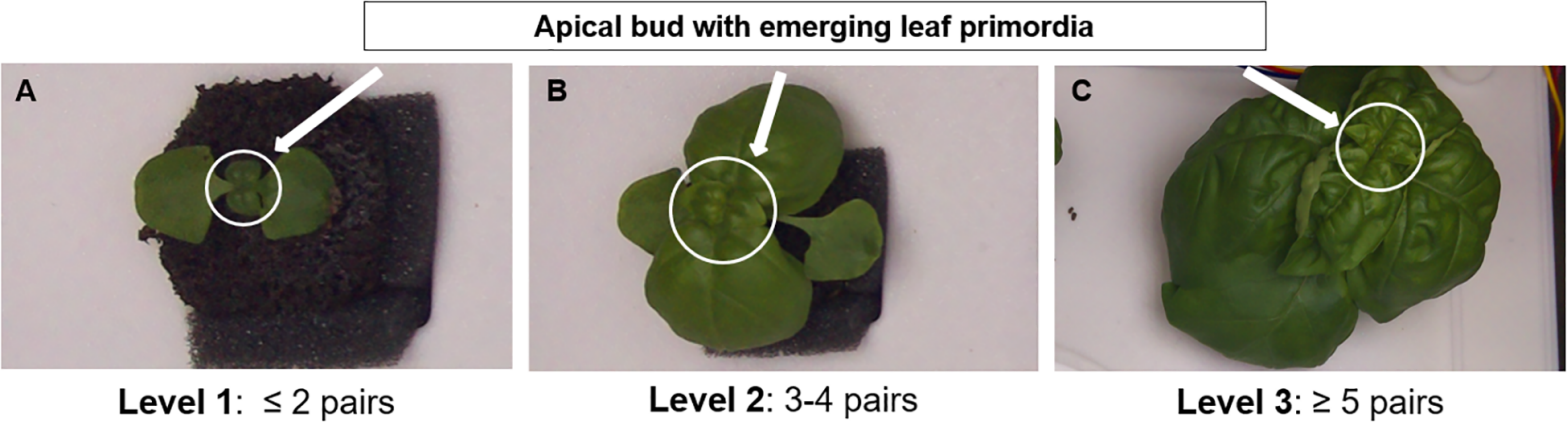
Representative examples of basil (*Ocimum basilicum* L.) growth stages based on the number of visible leaf pairs from top-view images: **(A)** Level 1 (≤ 2 pairs), **(B)** Level 2 (3–4 pairs), and **(C)** Level 3 (≥ 5 pairs). Arrows indicate the apical bud with emerging leaf primordia used as reference points for stage classification.

In this study, sweet basil (*Ocimum basilicum* L.) was selected as the model crop due to its high economic value and widespread cultivation in indoor and controlled-environment farming systems (Liaros et al., 2016). As a dicotyledonous species, basil exhibits a clear opposite phyllotaxy, producing paired leaves sequentially from the shoot apex (Dudai et al., 2020). Although overlapping foliage can hinder precise measurement of total leaf area as growth progresses, the shoot apex remains consistently visible, enabling reliable determination of developmental progress based on the number of newly emerged leaf pairs (**Figure 2A**). The leaf-pair number represents not only a morphological trait but also a physiologically meaningful indicator of growth. Each newly emerged leaf pair corresponds to a discrete developmental event accompanied by increased photosynthetic capacity, biomass accumulation, and overall plant vigor. Thus, this feature inherently reflects physiological growth dynamics without the need for direct measurement of chlorophyll content or stress indices. Furthermore, because basil continuously produces new leaf pairs at the upper apex, this trait effectively captures variations in individual growth rate that arise from environmental differences such as light intensity, temperature, or nutrient availability. Consequently, leaf-pair number provides a reproducible and environment-independent criterion for growth stage classification, offering a more physiologically grounded alternative to time-based approaches [e.g., days after sowing (DAS)]. Accordingly, top-view imaging was employed to non-destructively monitor basil growth status and detect leaf-pair development in multi-plant environments.

The Biologische Bundesanstalt, Bundessortenamt, and Chemical Industry (BBCH) scale is a standardized system designed to consistently encode phenologically similar growth stages across all monocotyledonous and dicotyledonous crop species. The principal growth stages are numerically represented from 0 to 9 in ascending order, enabling direct comparison among different plant species. In describing phenological development, the BBCH scale relies on clear and easily observable external traits that represent distinct physiological phases. Specifically, it defines early developmental stages of crops—particularly in dicotyledonous species—based on leaf number (Bleiholder et al., 2001). Building on this standard, the present study adopted the number of visible leaf pairs as a physiologically meaningful criterion for basil growth stage classification. This discrete trait is readily identifiable in top-view images and remains robust against visual distortions caused by overlapping or shading leaves.

For practical applicability, the growth process was subdivided into three levels: Level 1 (≤ 2 leaf pairs), Level 2 (3–4 leaf pairs), and Level 3 (≥ 5 leaf pairs) (**Figure 2**). This simplified classification framework facilitates efficient monitoring in cultivation environments, supports improved management and yield optimization, and provides a foundation for data-driven crop monitoring and automation.

### 2.3 Leaf-pair annotation and data preprocessing

To train the model on accurate leaf-pair counts, a preliminary manual labeling process was conducted for each basil plant. Labels were assigned sequentially from the top left to the top right and then from the bottom left to the bottom right within each image. All labeling was performed by a single researcher and verified three times to ensure consistency.

**Figure 3:**
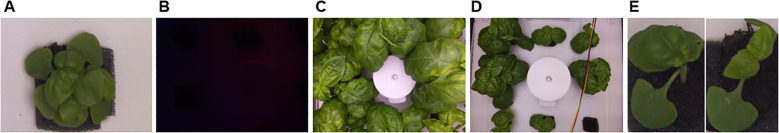
Examples of excluded images during preprocessing. **(A)** Multiple plants within a single pot; **(B)** Images captured during the dark period; **(C)** Plants more than half out of frame; **(D)** Non-germinated pots; **(E)** Plants with leaf removal history.

During this process, several problematic cases were identified and excluded (**Figure 3**). These included: (i) multiple seedlings germinating in a single pot (**Figure 3A**), where individuals completely overlapped and could not be separated, corresponding to images obtained before thinning; (ii) images captured during the dark period, producing entirely black frames (**Figure 3B**); (iii) plants extending more than half outside the field of view or obscuring the shoot apex (**Figure 3C**); and (iv) pots in which germination failed, yielding fewer than the expected eight individuals. Such cases were removed to maintain label reliability and avoid inconsistencies in ordering (**Figure 3D**). Another case was (v) plants with leaf abscission or removal, which were not excluded but instead handled during the counting process: if only one leaf of a pair was missing, the pair was still considered intact, whereas if both leaves were absent, the pair was counted as reduced by one (**Figure 3E**).

After this filtering process, the final training dataset comprised 2,169 images, including 334 from chamber 1, 310 from chamber 4, 237 from chamber 5, 294 from chamber 6, 357 from chamber 7, 330 from chamber 8, and 307 from chamber 9. No valid images remained from chambers 2 and 3 (**Table 2**).

**Table 2:** Number of collected images before and after filtering for each growth chamber.

Growth chamber no.	Number of images before filtering	Number of images after filtering
1	945	334
2	947	0
3	945	0
4	945	310
5	943	237
6	946	294
7	957	357
8	954	330
9	937	307
Total	8,519	2,169

### 2.4 YOLOv8-based plant detection and dataset preparation

All experiments were conducted in a Windows 11 environment equipped with an AMD64 Family 26 Model 68 Stepping 0 CPU (8 cores, 16 threads), 32 GB RAM, and an NVIDIA GeForce RTX 5070 GPU. The experimental codes were developed using Python 3.11 and PyTorch 2.7.1 (with CUDA Toolkit 12.8 support). We additionally utilized standard Python libraries for data processing and visualization, including NumPy, pandas, Pillow, scikit-learn, and OpenCV.

For automated recognition of individual plants within cultivation bed images, YOLOv8 was adopted to replace manual cropping, which is impractical for thousands of samples. Previous studies have demonstrated the effectiveness of YOLOv8 in mitigating complex background interference and improving downstream accuracy (Yang et al., 2023).

To train the detector, 217 images (approximately 10% of the dataset, evenly sampled across seven growth chambers) were annotated with bounding boxes indicating plant locations. To further reduce false positives, a small number of background-only images were included as negative samples, comprising ~1% of the dataset (Ultralytics, 2023). The final training set consisted of 219 images, which were divided into training, validation, and test subsets in a 70:20:10 ratio (153:44:22).

### 2.5 Preprocessing strategies to reduce YOLO skip rate

Images were retained as training data only when YOLO successfully detected exactly eight plants; otherwise, the images were discarded to avoid mismatched labels. However, this strict criterion initially resulted in a high skip rate, leading to substantial data loss and potential performance decline (**Tables 3A, B**).

**Table 3:** Number of skipped images under different preprocessing conditions for each growth chamber.

Growth chamber no.	(a) Skips without negative samples and preprocessing	(b) Skips without preprocessing	(c) Skips after applying F1 confidence threshold	(d) Skips after applying F1 confidence threshold + nested box removal
1	2	2	1	1
4	4	2	1	0
5	22	13	12	12
6	24	12	3	3
7	34	23	11	9
8	49	20	3	1
9	38	1	1	1
Total	173	73	32	27

Analysis of skipped cases revealed two main issues: (i) single plants were occasionally recognized as multiple objects, and (ii) background regions were misidentified as plants. To mitigate these errors, two preprocessing strategies were introduced. First, based on the F1–confidence curve, a threshold of 0.706 was applied, retaining only bounding boxes above this value. Second, when a bounding box was nested within another, the smaller box was removed using a 10-pixel tolerance to resolve overlapping detections.

These steps substantially reduced the skip rate (**Tables 3C, D**), enabling construction of a more accurate and stable training dataset.

### 2.6 Automated labeling and dataset construction pipeline

**Figure 4:**
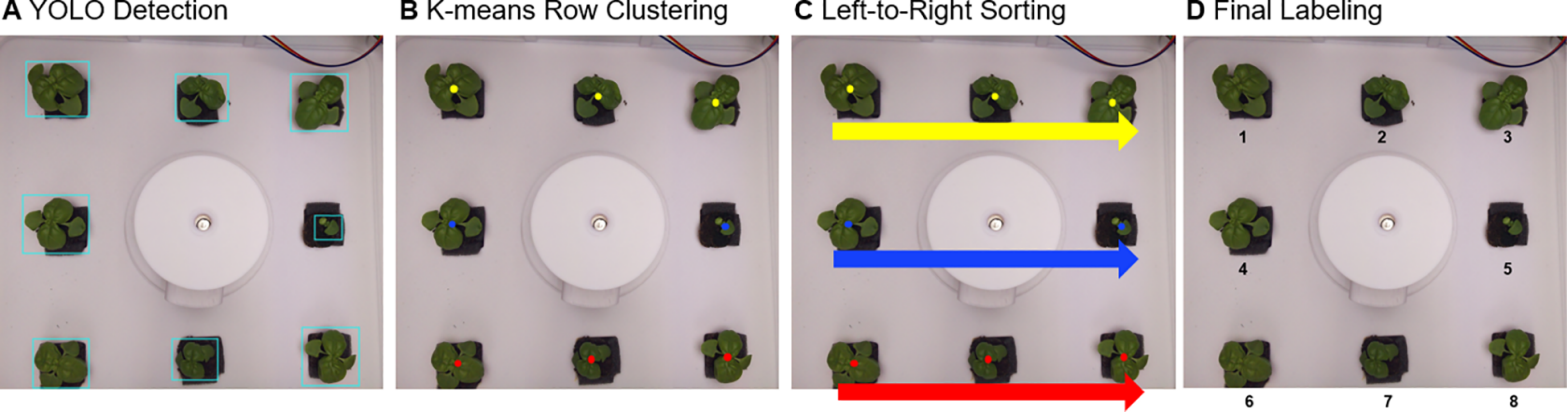
Automated labeling pipeline for basil plants: **(A)** YOLOv8 detects individual plants, **(B)** the detected plants are grouped into rows using K-means clustering, **(C)** plants within each row are sorted from left to right, and **(D)** sequential numbering is assigned for consistent labeling.


If eight bounding boxes were successfully detected through the above process (**Figure 4A**), each object had to be arranged according to a predefined labeling order. To achieve this, the center coordinates of each bounding box were calculated from the YOLO output coordinates ( $x_{1},\;y_{1},x_{2},y_{2}$):

$$c_{x}=\frac{x_{1}+x_{2}}{2\;},\;\;c_{y}=\frac{y_{1}+y_{2}}{2\;}$$

First, based on the vertical position ( $c_{y}$) of the center coordinates, K-means clustering (k=3) was applied to classify plants into three rows: top, middle, and bottom (**Figure 4B**). Within each row, plants were then sorted from left to right according to their horizontal positions ( $c_{x}$) (**Figure 4C**). The final labeling sequence was thus defined as: top row (left → right) → middle row (left → right) → bottom row (left → right) (**Figure 4D**).

**Figure 5:**
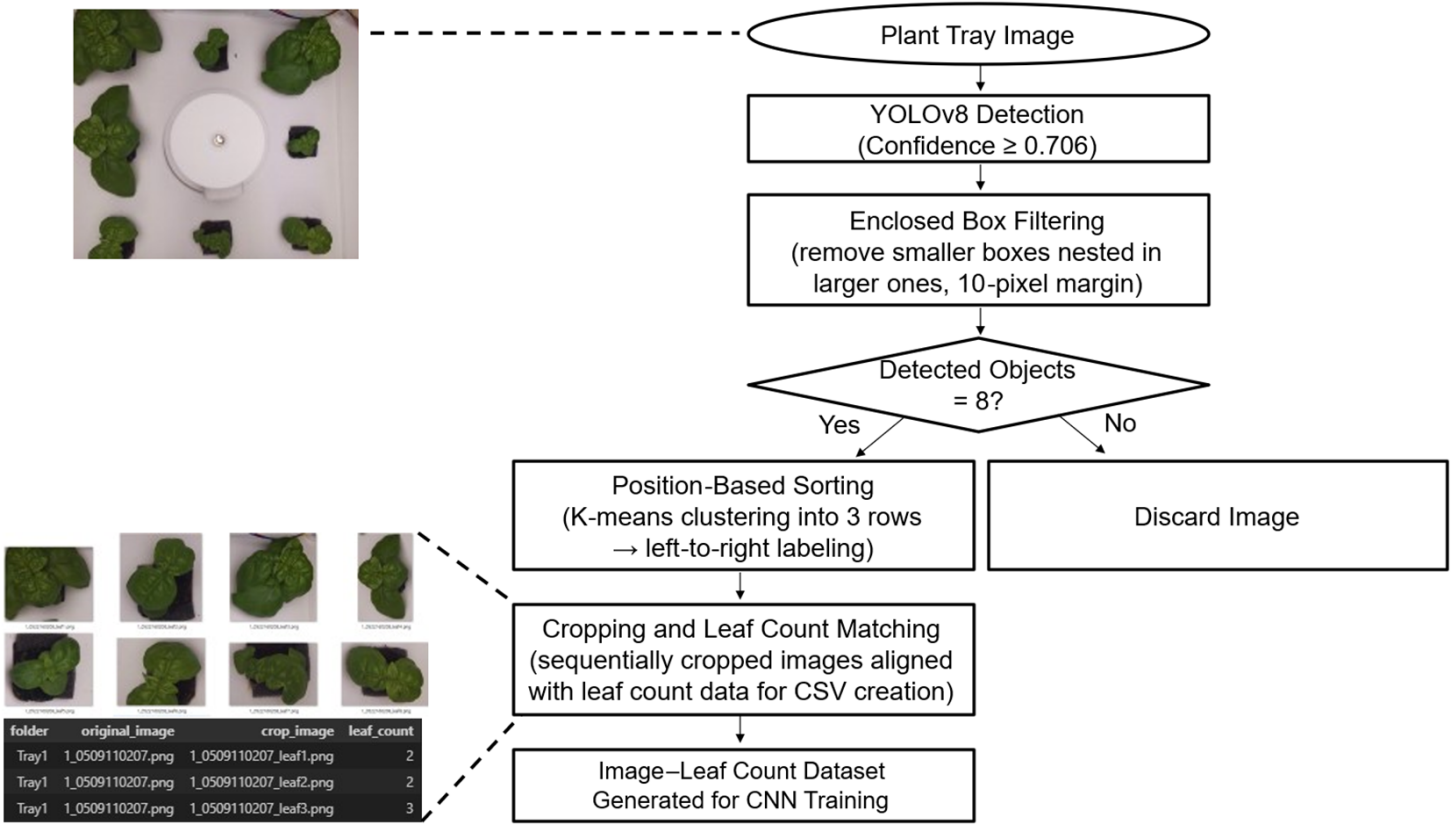
Automated pipeline for generating cropped plant images with matched leaf count labels for CNN model training, using YOLOv8 detection and preprocessing.

After labeling, each bounding box region was cropped and paired with the corresponding leaf-pair count to generate image–label datasets in CSV format for Convolutional Neural Network (CNN) training (**Figure 5**).

An alternative approach would be to assign labels based on predefined coordinates; however, this method is highly sensitive to variations in camera angle or tray placement, limiting its applicability. In contrast, the YOLO–K-means strategy employed here ensured consistent ordering, demonstrated robustness to environmental variation, and provided flexibility for extension to large-scale cultivation systems such as plant factories.

### 2.7 Regression-based convolutional neural network for leaf-pair estimation

Through the YOLO-based automated labeling pipeline, 12,871 cropped image–leaf pair samples were constructed from 1,952 valid images, excluding those used for YOLO training or containing occluded individuals with invisible shoot apices. The dataset was split into training, validation, and test sets in a 70:20:10 ratio, comprising 9,009, 2,574, and 1,288 samples, respectively, with a fixed random seed to ensure reproducibility.

A custom CNN model was developed based on the ResNet-18 architecture pre-trained on ImageNet. The classification head was replaced with a fully connected layer, and dropout and activation functions were added to enable continuous leaf-pair prediction. A regression approach was selected over categorical classification because it penalizes errors proportionally to their magnitude, thereby supporting more precise convergence (Wang et al., 2022; Widrow and Lehr, 2002). Predicted values were then post-processed by rounding to the nearest integer and mapping to predefined growth stages.

### 2.8 Hyperparameter optimization with Optuna

Hyperparameter optimization was performed using Optuna to improve model performance. Previously, researchers had to adjust hyperparameters in a cumbersome manner manually, and such manual and static search approaches were inefficient and limited in terms of objectivity and resource efficiency (Akiba et al., 2019). In this study, these limitations were addressed by applying Optuna-based automated hyperparameter optimization, thereby aiming to achieve efficient and systematic optimization. The search space included key parameters across data preprocessing, model architecture, and training. For data augmentation, variations in brightness, contrast, saturation, hue (ColorJitter), flipping, and rotation were optimized to simulate changes in lighting and viewing conditions. For the architecture, activation functions (ReLU, LeakyReLU, GELU) and dropout rates were explored, while the training process considered learning rate, optimizer type, weight decay, early stopping patience, loss function, and scheduler settings. Image augmentation is used to enhance the performance of deep learning models and prevent data overfitting by increasing the quantity, diversity, and quality of images (Boissard et al., 2008; Li et al., 2020). In agricultural research, the most common and cost-effective techniques for expanding dataset size are flipping and rotation, while brightness/color adjustments, blurring, and sharpening are additionally applied to improve the generalization performance of models under complex backgrounds or varying illumination conditions (Antwi et al., 2024).

Optimization was carried out using Optuna’s Tree-structured Parzen Estimator (TPE) sampler with MedianPruner for early stopping of low-performing trials. A total of 30 trials were conducted, yielding an optimized configuration based on empirical performance rather than manual selection.

### 2.9 Huber loss function for robust regression

The Huber loss function is a combination of the advantages of r. For small errors, it applies a squared loss to promote precise convergence and enhance fine-grained predictions, while for large errors, it applies an absolute loss to mitigate the instability in training caused by outliers (Huber, 1964). The Huber loss function is defined as follows:

$Huber\;Loss=\sum_{n=1}^{N}l_{n},\;$ where


$$l_{n}\;=\;\{\begin{array}{c} \;\frac{1}{2}{\left(x\_n\;-\;y\_n\right)}^{2}\;\;\;\;\;\;\;\;\;\;if\;\left|{\text{x}}_{\text{n}}-{\text{ y}}_{\text{n}}\right|<\;\delta \;\\ \delta \left(\left|{\text{x}}_{\text{n}}-{\text{ y}}_{\text{n}}\right|-\frac{1}{2}\text{δ}\right)\;\;\;\;\;\;\;otherwise\; \end{array}$$

Here, $\;x_{n}$​ denotes the predicted value, $y_{n}$​ the actual value, and δ the threshold that determines the transition between the squared loss and the absolute loss. Since the dataset in this study employed manually counted leaf pair numbers as labels, a certain level of noise could have been introduced during the labeling process. In particular, the emergence of leaf pairs is characterized by a gradual rather than strictly discrete formation, making it prone to errors caused by subjective judgment. Under such conditions, the Huber loss function is advantageous because it is robust to outliers and helps mitigate training instability arising from noisy data (Meyer, 2021).

The Huber loss function has also been widely applied in the agricultural domain. Dwaram and Madapuri (2022) employed Huber loss for training an LSTM-based crop yield prediction model and achieved the best performance among the tested settings with an accuracy of 98.02% and an F1-score of 98.97% (Dwaram and Madapuri, 2022). Guo et al. (2023) found that, in estimating carbon content using remote sensing, the Huber loss function outperformed MAE, MSE, and Smooth L1 by providing robustness to outliers and higher inference accuracy (Guo et al., 2023).

### 2.10 Integrated inference pipeline for growth stage classification

**Figure 6:**
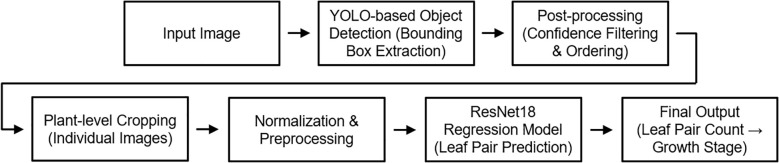
Integrated inference pipeline for plant growth stage classification. YOLOv8 detects individual plants, ResNet-18 regression estimates leaf-pair counts, and predictions are converted into discrete growth stages.

An integrated inference pipeline was developed to automatically determine the growth stage of individual plants from growth chamber images (**Figure 6**). The pipeline comprised three steps: (i) detection of individual plants using YOLOv8, followed by preprocessing with overlapping box removal and coordinate-based ordering; (ii) prediction of leaf-pair counts from cropped regions with a ResNet-18 regression model; and (iii) conversion of continuous predictions into integer values and classification into growth stages (Levels 1–3).

Final outputs included visualization images, with bounding boxes annotated by predicted stages and leaf-pair counts, as well as CSV files for quantitative analysis. By integrating the proposed detection, preprocessing, and regression steps, this pipeline provides a practical framework for automated growth monitoring in controlled cultivation environments.

## 3 Results and discussion

### 3.1 Detection performance of the YOLOv8 model

**Figure 7:**
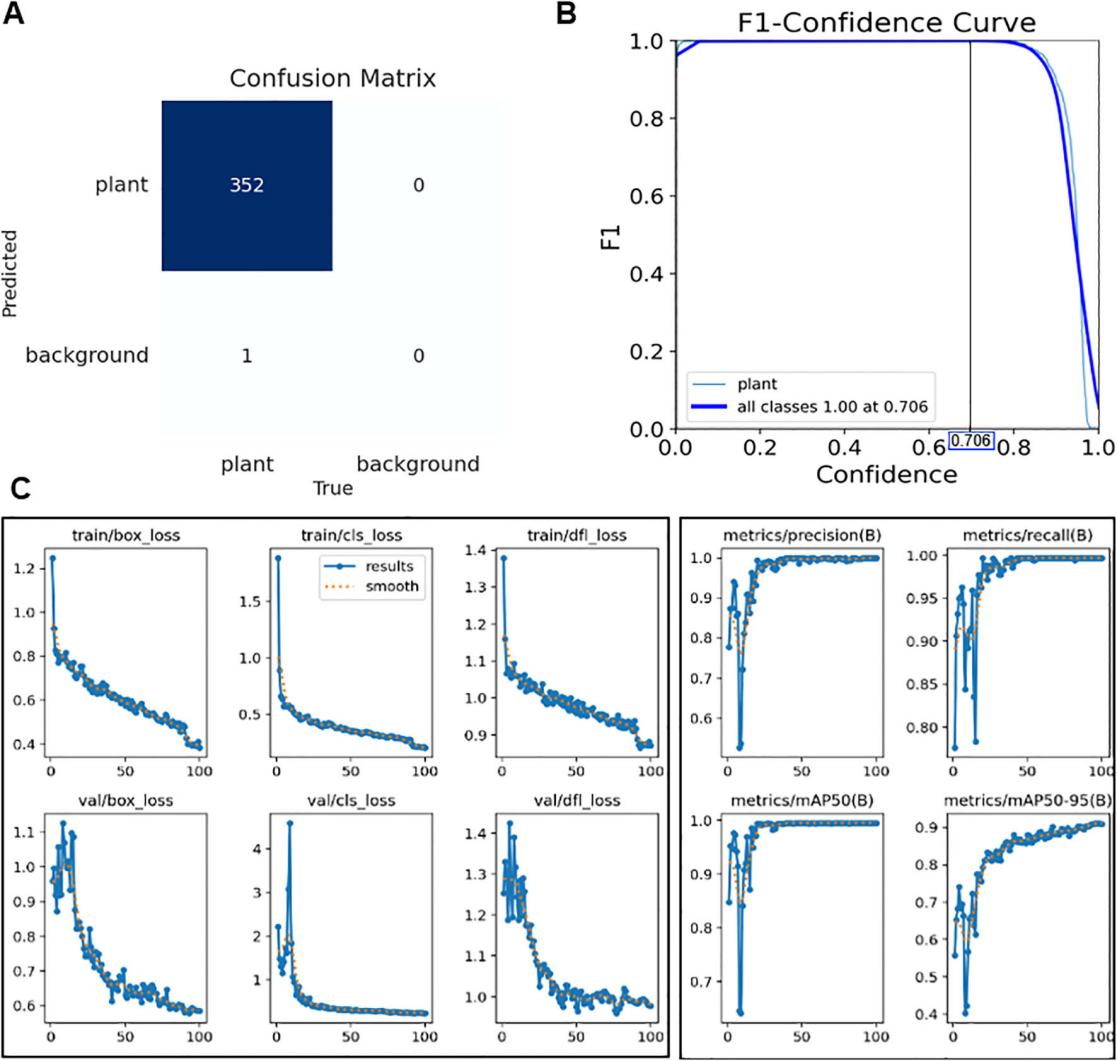
**(A)** Confusion matrix for the validation set, showing 352 true positives, 0 false positives, and 1 false negative. **(B)** F1–confidence curve illustrating the optimal confidence threshold that maximizes the F1-score. **(C)** Training and validation curves for box loss, classification loss, distribution focal loss, precision, recall, and mAP@0.5/0.5–0.95 over 100 epochs (x-axis: epoch, y-axis: corresponding loss or metric value).

The confusion matrix (**Figure 7A**) summarizes detection outcomes for the 20% validation set (44 images with approximately eight plants per image), which contained 353 annotated plant instances. The model correctly detected 352 instances with no false positives and only one missed plant, corresponding to Precision = 1.000 and Recall = 0.997.

The YOLOv8 detector was trained on the training subset derived from 219 annotated images for 100 epochs, during which all loss terms (box_loss, cls_loss, and dfl_loss) steadily decreased and converged, while precision, recall, and mean Average Precision (mAP) metrics consistently improved (**Figure 7C**). This indicates that the model was well-optimized without signs of overfitting. The F1–confidence curve confirmed stable performance, maintaining an F1-score of 1.000 up to a confidence threshold of 0.706, beyond which the score declined (**Figure 7B**). On the final validation set, the detector achieved an mAP@0.5 of 0.995 and an mAP@0.5:0.95 of 0.905, with both precision and recall reaching 1.000. **Figures 7A-C** demonstrate that YOLOv8 achieved stable convergence without overfitting and maintained perfect detection accuracy across a wide confidence range.

These results suggest that the model could detect all plants in the growth chamber without false positives or false negatives, thereby providing highly reliable cropped images for subsequent leaf-pair regression and growth stage classification. The absence of misdetections at this stage is particularly critical, since errors in detection would directly propagate to later stages of the pipeline and undermine stage classification accuracy.

These improvements were supported by systematic preprocessing strategies, including negative sample incorporation and nested box removal, which reduced skip rates and enhanced detection stability. The determination of an optimal confidence threshold also highlights the practical utility of the model, as it allows fine-tuning of detection sensitivity according to cultivation needs—for example, prioritizing recall in early growth monitoring versus precision in harvest-stage assessments. From an application perspective, this robustness is particularly important in dense multi-plant images, where even minor misdetections could propagate errors to later stages of classification. Nevertheless, further validation under more heterogeneous conditions, such as variable lighting or field environments, will be essential to confirm the broader generalizability of the detector.

### 3.2 Hyperparameter optimization results for CNN regression

The optimal combination of hyperparameters automatically selected through the Optuna framework was as follows. The learning rate was set to a very small value of 3.67×10^-5^, and the AdamW optimizer was used together with a weight decay of 4.81×10^-4^ to prevent overfitting and ensure stable convergence. The dropout rate was set to 13.4%, which was relatively low, thereby maintaining sufficient training flexibility without excessive regularization. The early stopping condition was configured with a patience of 7 epochs, reducing unnecessary iterations while allowing convergence without performance degradation.

For data augmentation, variations were applied using ColorJitter, including brightness (± 10.6%), contrast (± 24.5%), saturation (± 36.6%), and hue (± 5.6%). In addition, horizontal flipping was applied with a probability of 26.3%, and random rotations were allowed up to ±10°. In this study, the selected augmentation strategy was particularly effective because it realistically simulated lighting fluctuations and viewpoint changes that frequently occur in indoor cultivation systems, thereby reinforcing the robustness of the model under practical farming conditions.

This augmentation strategy effectively enhanced both the diversity of the experimental data and the generalization capability of the model. The activation function selected was LeakyReLU, which alleviates the dead neuron problem of ReLU by maintaining a small gradient for negative input values, thereby preventing learning stagnation. No learning rate scheduler was used, indicating that the learning rate itself was sufficiently small and stable throughout training.

These results underscore the importance of systematic hyperparameter tuning in agricultural vision applications. Whereas prior studies often relied on heuristic parameter choices, automated optimization enabled a configuration that balanced convergence speed, generalization, and robustness to noisy labels. In practice, this approach supports reliable deployment in diverse cultivation settings, where environmental variability can otherwise degrade model performance. Moreover, the automated search process improved reproducibility by reducing dependence on subjective trial-and-error, ensuring that the derived configuration reflected data-driven evidence rather than manual intuition. From an application perspective, such optimized settings are highly advantageous for large-scale deployment in plant factories or smart farms, where environmental variability and computational constraints demand models that are both accurate and efficient.

### 3.3 Performance evaluation of the proposed growth stage model

**Figure 8:**
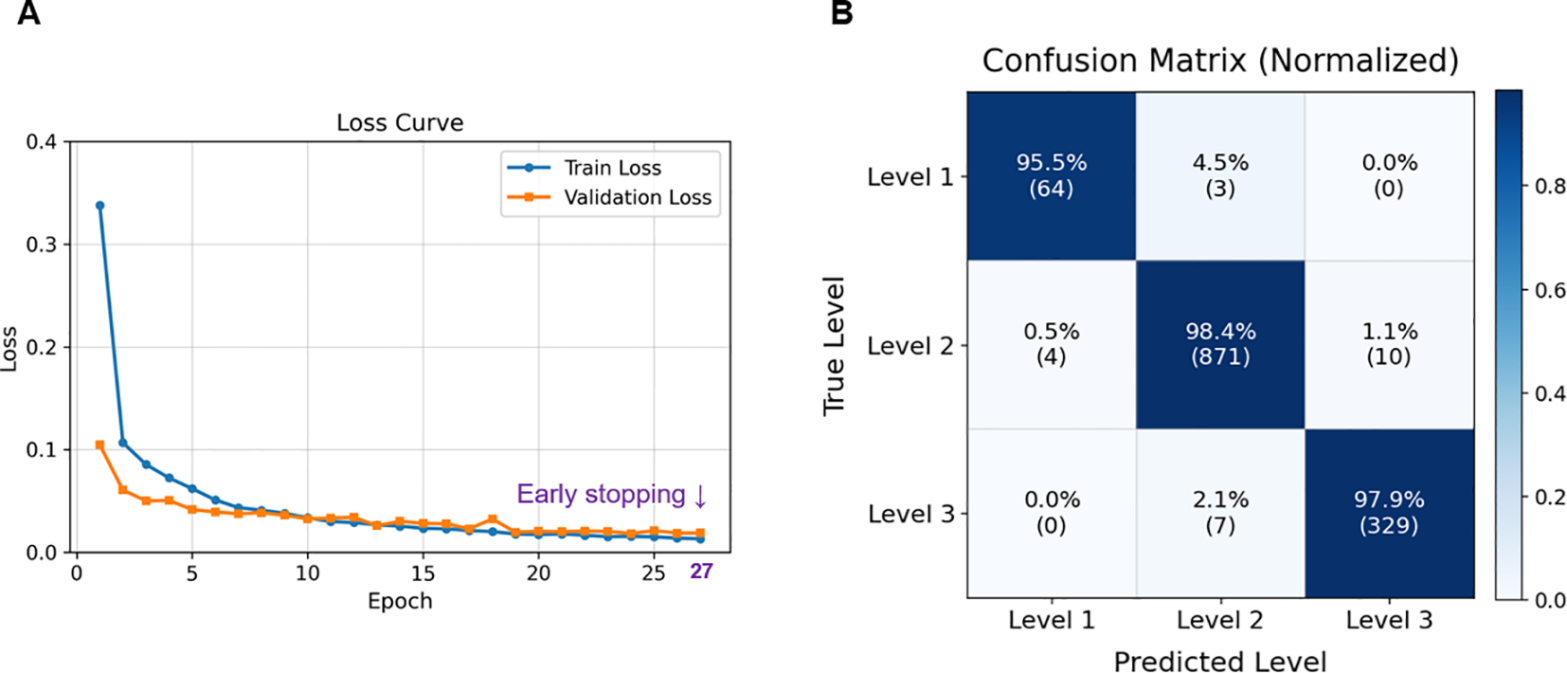
Model training and classification performance. **(A)** Training and validation loss curves showing stable convergence, with early stopping triggered at the 27th epoch to prevent overfitting; **(B)** Normalized confusion matrix for Level 1–3 classification on the test set, indicating high prediction accuracy across all levels.

The regression model demonstrated stable convergence, with both training and validation losses decreasing smoothly and early stopping triggered at the 27th epoch (**Figure 8A**). This stability reflects effective regularization, and the use of the Huber loss function likely contributed to reducing the influence of noisy labels while preserving sensitivity to fine-grained leaf-count differences. The normalized confusion matrix confirmed high predictive accuracy across all growth stages, with Level 2—representing a transitional phase of leaf development—achieving the highest accuracy at 98.4% (**Figure 8B**).

Quantitative evaluation further demonstrated the reliability of the proposed approach. On the fixed test set, the model achieved a Mean Absolute Error (MAE) of 0.13, a Mean Squared Error (MSE) of 0.04, a Root Mean Squared Error (RMSE) of 0.20, and a Coefficient of determination (R²) of 0.96, while stage classification accuracy and weighted F1-score reached 98.1% and 0.98, respectively. These values substantially outperform those of earlier studies. For example, Yang et al. (2025) employed U-Net–based segmentation to extract individual plants from multi-plant images and performed BBCH stage classification. However, the model performance based solely on images remained around 73%. To improve accuracy, they incorporated additional features such as area, perimeter, pixel count, and inter-image similarity derived from the segmented masks. Nevertheless, such trait-based features are highly sensitive to changes in camera height or imaging conditions and were limited to images captured under white light, thereby reducing reproducibility and generalizability. Furthermore, the pipeline involved excessive complexity and multiple modules, with segmentation and classification operating separately, which limits its applicability for real-time field deployment (Yang et al., 2025).

**Figure 9:**
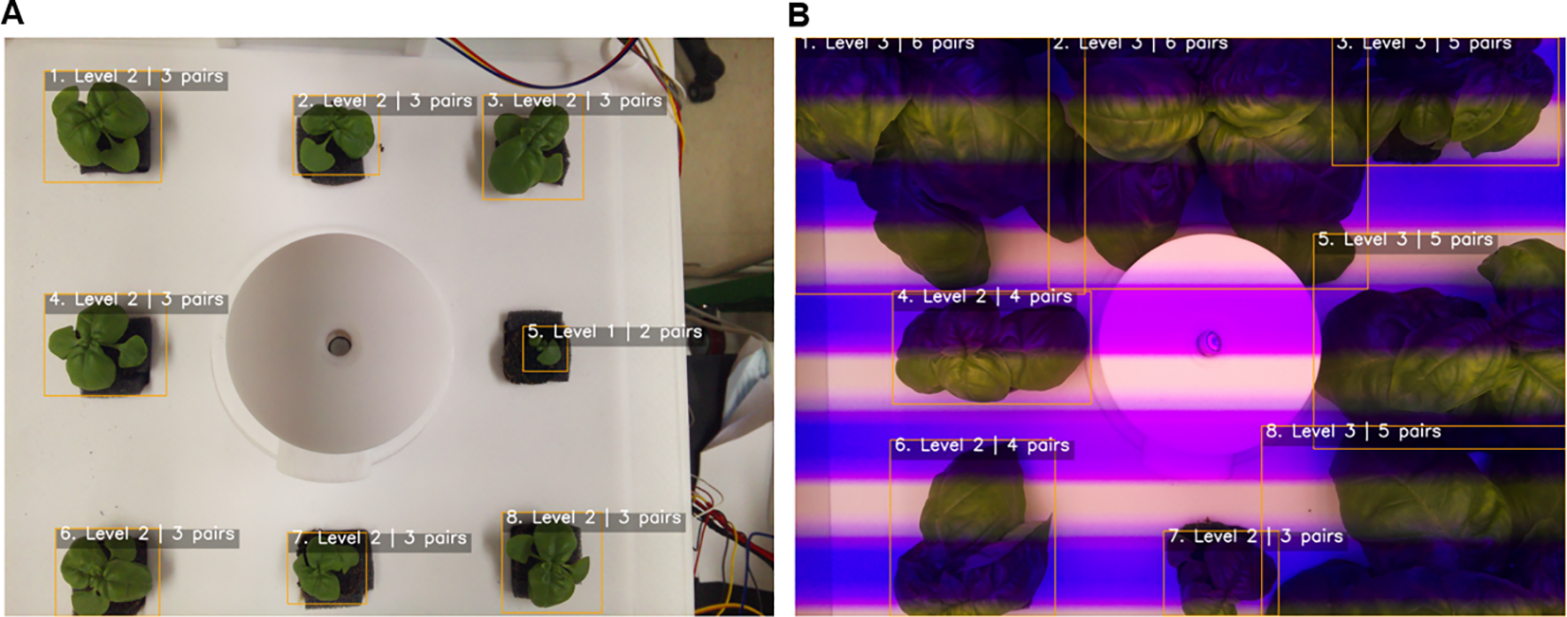
Automated growth stage classification of basil plants. **(A)** Cultivation bed 1, showing detection and classification of individual plants based on leaf-pair counts; **(B)** Cultivation bed 4, demonstrating stable multi-plant detection and classification under artificial lighting. Bounding boxes denote individual plants, and labels indicate the predicted growth stage (Level 1–3) and leaf-pair number.

The robustness of the framework was further validated through 5-fold cross-validation, which yielded consistent results (MAE 0.12 ± 0.01, R² 0.97 ± 0.004). This consistency indicates that the model is not overly dependent on specific data subsets and can generalize well to unseen samples. In addition, the consistently high performance across folds demonstrates that the model is resilient to data imbalance and noise, further reinforcing its robustness. Importantly, the ability to detect and classify multiple individuals within a single frame directly overcomes the scalability limitations of prior single-plant approaches. Representative examples are shown in **Figure 9**, where the pipeline accurately identified and classified basil plants under both natural and artificial lighting conditions, demonstrating resilience to variation in imaging environments. In **Figure 9A**, all eight individuals in the cultivation bed were correctly detected and assigned growth stages, with shoot apices clearly visible and minimal occlusion, allowing stable classification at early growth stages. In contrast, **Figure 9B** illustrates more complex conditions, including overlapping leaves, partial occlusion, and strong color distortion caused by LED lighting; nevertheless, the pipeline successfully recognized individual plants and determined leaf-pair numbers and growth stages with high reliability. These results highlight the robustness of the framework in dense cultivation environments, where visual interference is common, and underscore its practical applicability in smart farming systems. Unlike prior studies that often relied on single-plant images or used only the largest contour from multi-plant frames, the present approach enables instance-level detection and classification of all individuals simultaneously, ensuring scalability for real cultivation settings.

From an application standpoint, achieving over 98% accuracy in growth stage classification highlights the potential of this framework as a core component of precision agriculture systems. Reliable and automated stage detection could support fine-tuned scheduling of nutrient delivery, adaptive control of lighting and climate, and optimization of harvest timing. By grounding stage classification in physiologically meaningful traits—namely leaf-pair number—the proposed method strengthens the reproducibility of plant research while advancing the practicality of smart farming technologies. Nevertheless, as the current experiments were conducted in controlled environments, future validation under larger datasets, diverse crop species, and field conditions will be necessary to confirm broader applicability and generalizability.

### 3.4 Future directions and potential applications

Future research should prioritize the development of lightweight models and the validation of inference performance in real cultivation systems. Although the current framework achieved high accuracy under controlled conditions, its computational demands may limit scalability in commercial applications. By reducing inference time, lightweight architecture can facilitate real-time integration with automated crop monitoring and environmental control systems, which is essential for the commercialization of smart agriculture. Previous approaches, such as complex multi-stage pipelines, also suffered from slow processing speeds, making them unsuitable for large-scale field monitoring or real-time control. Therefore, for applications in controlled environments such as plant growth chambers or plant factories, the development of lightweight models capable of operating reliably and efficiently even on low-power devices is indispensable.

Another promising direction is the incorporation of temporal modeling. While the proposed model effectively captures instantaneous growth states, it is limited in reflecting the continuous progression of growth and stage transitions that are critical in real cultivation environments. Integrating sequence-based architectures such as LSTM would enable dynamic tracking of these transitions and provide richer temporal information, thereby improving predictive accuracy for tasks such as nutrient scheduling and harvest timing.

Extension to other dicotyledonous leafy vegetables and herbs, such as lettuce and kale, will also be critical for broad applicability. While basil served as a representative model in this study, crop-specific growth traits may require tailored optimization before integration into a generalized multi-crop framework. Likewise, scaling the approach to diverse cultivation environments—including plant factories, smart farms, and systems with larger plant populations—will test the robustness of the pipeline under heterogeneous conditions.

Finally, integrating image-based phenotypic data with environmental information such as light spectra, temperature, and humidity will be essential to further improve accuracy and stability. A unified data-driven framework that links growth stage detection directly to environmental control would enable precise, stage-specific interventions, advancing both the scientific reproducibility of crop growth studies and the practical realization of sustainable smart farming.

## 4 Conclusion

This study introduced a novel pipeline for instance-level growth stage classification of basil using top-view multi-plant images, addressing fundamental limitations of previous research that relied on time-based staging or single-plant imaging. By integrating YOLOv8-based object detection, K-means–based coordinate ordering, and a ResNet-18 regression model, the proposed “detection–ordering–regression–staging” framework achieved state-of-the-art performance. The YOLOv8 detection module achieved mAP@0.5 = 0.995, mAP@0.5:0.95 = 0.905, Precision = 1.000, and Recall = 0.997, confirming near-perfect instance-level localization across all plants in the validation set and providing reliable cropped inputs for subsequent regression and classification. As a result, the overall framework attained MAE = 0.13, RMSE = 0.20, and R² = 0.96, with a growth stage classification accuracy of 98.1% (F1 = 0.98).

The novelty of this work lies in its ability to perform automated, non-destructive, and physiology-based stage classification that captures the developmental progression of individual plants directly from multi-plant images, a task that prior approaches could not reliably accomplish. Unlike conventional indices such as biomass or leaf area, which are highly sensitive to imaging conditions, traits such as total leaf area or canopy coverage require multi-angle imaging and are easily distorted by leaf drooping, occlusion, or camera perspective differences. In contrast, the number of leaf pairs at the shoot apex provides a discrete, BBCH-aligned, and physiologically meaningful criterion that remains clearly visible from top-view images regardless of growth density or camera placement. This trait inherently reflects photosynthetic capacity, biomass accumulation, and overall growth dynamics, enabling reproducible classification under realistic cultivation environments without the need for labor-intensive manual observation or controlled image preprocessing.

Notably, the framework demonstrated scalability: although developed in an eight-pot fixed-bed system, the flexible detection–ordering logic can be adapted to different layouts without retraining, ensuring applicability to diverse cultivation facilities. Because the framework relies on a visually stable and physiologically grounded trait rather than time-dependent or angle-sensitive measures, it can robustly accommodate environmental variability such as light intensity, temperature, or nutrient differences, ensuring consistent performance even under heterogeneous imaging conditions. Cross-validation confirmed consistent performance, underscoring the robustness of the pipeline for large-scale deployment.

In summary, this study is the first to establish an integrated and fully automated workflow that couples object detection with physiology-driven regression modeling for growth stage determination in multi-plant settings. Beyond reducing labor requirements, the framework has strong potential as a core technology for precision agriculture, enabling stage-specific environmental control, adaptive nutrient and water management, yield forecasting, and early stress detection. By providing a simple yet reliable visual indicator optimized for camera-based automation, the proposed system bridges morphological observation with physiological interpretation, offering both scientific insight and operational practicality for intelligent cultivation systems. Future research should extend this approach to other leafy vegetables and diverse cultivation systems, while incorporating lightweight and temporal modeling strategies to further advance commercial adoption in smart farming.

## Data Availability

The raw data supporting the conclusions of this article will be made available by the authors, without undue reservation.
